# Creatinine Kinetic Modeling to Estimate Residual Kidney Creatinine Clearance in Patients Being Hemodialyzed Once or Twice Per Week

**DOI:** 10.1111/sdi.70009

**Published:** 2025-10-03

**Authors:** John T. Daugirdas, Piergiorgio Bolasco

**Affiliations:** ^1^ University of Illinois at Chicago College of Medicine Chicago Illinois USA; ^2^ Working Group for the Conservative Treatment of Chronic Kidney Failure of the Italian Society of Nephrology Rome Italy

## Abstract

**Background:**

Knowledge of residual kidney function is potentially useful in patients receiving hemodialysis for risk stratification, adjusting the dialysis prescription, and early identification of renal function recovery. However, periodic urine collection is problematic. We examined the potential of predicting residual kidney creatinine (water) clearance (KrCrW) without urine collection using a creatinine kinetic model, which allows KrCrW to be estimated based on previously measured or anthropometrically estimated creatinine generation rate (GCr), volume of distribution (VdCr), and measured predialysis serum creatinine.

**Methods:**

Studies were done in 12 patients receiving once weekly hemodialysis and 12 other patients being dialyzed twice a week in whom KrCrW was measured by collection of urine. GCr and VdCr were taken either from the modeling outputs or were estimated from anthropometric values.

**Results:**

The mean modeled GCr was 1091 ± 377 (SD) mg/day, similar to the value predicted by an anthropometric equation suggested by Ix et al. (1198 ± 304). The mean kinetically modeled VdCr was 22.7 ± 2.4 L, somewhat lower than expected. The KrCrW from urine collection was 7.43 ± 4.07 mL/min. Predicted KrCrW from modeled GCr, modeled VdCr, and measured predialysis serum creatinine was similar (7.35 ± 4.01, *r* ^2^ = 0.987) with an average error less than 1%. When anthropometric estimates of GCr and VdCr were used as inputs, the mean modeled KrCrW was somewhat higher (8.66 ± 4.27, *y* = 1.09*x*, *R* ^2^ = 0.585) and the mean error was 1.23 ± 2.6 mL/min.

**Conclusions:**

Residual kidney creatinine clearance (KrCrW) can be estimated in patients receiving one or two dialysis treatments weekly based on creatinine kinetic modeling. Using anthropometric estimates of GCr and VdCr in the modeling equations yields similar values of KrCrW to those when modeled GCr and VdCr inputs are used, but with a substantial error. A strategy of using a baseline modeled values of GCr and VdCr for future KrCrW change prediction may be promising, but the stability of GCr over time needs to be confirmed.

## 1 Introduction

Recent literature has focused attention on the importance of residual kidney function in patients receiving dialysis [1]. The usual method of determining GFR is to collect 24 h urine and analyze it for creatinine. In the ESKD population, both creatinine and urea are commonly analyzed, and GFR is taken to be the average of the two measurements. However, apart from expense, there are problems involved in the logistics of proper urinary collection, involving patient understanding and recording of start and end collection times, as well as forgetting to urinate into the collection bottle with every void during the collection period [2]. In the calculation of creatinine clearance, if the generation rate, the volume of distribution, and the serum concentration are precisely known, the clearance can be calculated mathematically without the need for measurement of urine excretion. When modeling dialysis patients, a relatively small extrarenal creatinine clearance and, of course, the dialyzer clearance must be taken into consideration, but the principle is the same: If the generation rate, the dialysis and extrarenal clearances, and the creatinine volume of distribution are known, the residual renal clearance can be determined from the predialysis serum creatinine level without the need to collect and assess urine excretion.

In creating the required creatinine kinetic model for dialysis patients, the required calculations are complicated by the fact that dialysis schedules are almost invariably asymmetric, and when the predialysis serum creatinine sample is obtained just prior to a dialysis treatment, the number of nondialysis days during the previous interdialytic interval, during which generated creatinine accumulates, differs depending on the dialysis schedule. During the past 15 years, one of us has developed a series of kinetic models that can be used to estimate either clearances or serum concentrations of solutes in patients receiving maintenance hemodialysis [3]. A model focusing on creatinine [4] was validated against data obtained in the course of the HEMO study and was designed to calculate the creatinine generation rate (GCr) and creatinine volume of distribution (VdCr) based on inputs of pre‐ and post‐dialysis serum creatinine levels, residual renal creatinine (water) clearance (KrCrW) as well as dialyzer treatment and schedule data. A second, “what‐if” model [3, 5] uses inputs of GCr, VdCr, and KrCrW in order to calculate the expected pre‐ and post‐dialysis serum creatinine levels for a specified dialysis day of the week. The new variant of this creatinine kinetic modeling program, composed for the purpose of the present analysis, is designed to calculate KrCrW based on inputs of GCr and VdCr, and predialysis serum creatinine, along with dialysis treatment and schedule information. For this study, we used this new KrCrW program to calculate the modeled KrCrW and compared this with the measured KrCrW from urine collection data.

## 2 Methods and Design

Twenty‐four patients undergoing hemodialysis with schedules of fewer than three treatments per week were analyzed. Twelve (age: 70.8 ± 13.2 years, nine male and three female) of these patients were being dialyzed once a week, and another 12 (age: 68.0 ± 7.6 years, eight male and four female), were receiving two treatments weekly, either on a Monday–Friday or Tuesday–Saturday schedule. For each patient, the predialysis urine collection was handled in two ways. Urine was collected during the complete interdialytic interval preceding the modeled dialysis treatment in daily aliquots. Creatinine clearance was calculated based on the urine aliquot collected during the most recent 24‐h period prior to dialysis, and also from all of the urine samples collected during the interdialytic interval that were combined (mixed together). KrCrW was calculated in the usual fashion. The per minute average creatinine excretion rate was determined as the average urine creatinine concentration during the collection period multiplied by the urine volume divided by the number of minutes in the collection period. This was then divided by the time‐averaged serum water concentration of creatinine during the collection period; this serum concentration was determined by the creatinine kinetic modeling program, which develops a minute‐by‐minute record of the steady‐state serum creatinine values over an entire week. The “urea solute solver, version 2.14” and “creatinine solute solver, version 1.23” programs, available at ureakinetics.org [3], were used to model the index dialysis treatment. The dialyzer urea and creatinine clearances were estimated using the Michaels equation from adjusted values of in vitro K_0_A for urea. This in vitro value was multiplied by 0.537 for urea and by 0.360 for creatinine, to adjust for the in vivo situation and the lesser clearance for creatinine compared with urea. The Michaels equation was then used, based on inputs of blood water or plasma water flow and dialysate flow to calculate a diffusive dialyzer clearances for urea and creatinine, respectively, and then these clearances were adjusted for each dialysis treatment based on the ultrafiltration rate during that treatment, as previously described [6].

Both urea and creatinine solvers are based on the same assumptions, that urea or creatinine is removed from a proximal pool, which is replenished from a distal pool at a rate that depends on the intercompartmental clearance that is a multiple of body weight [3, 6].

In the creatinine solute solver program, the inputs are the KrCrW, the pre‐ and post‐dialysis serum creatinine levels, the dialysis schedule, and the dialysis day on which the pre‐ and post‐dialysis blood samples are drawn. The start and end times of the urine collection are also an input, along with the urine volume and urine creatinine concentration. The modeling program develops a weekly profile of the serum creatinine concentration based on an initial assumption for GCr, which is then adjusted by iteration until the modeled and measured predialysis serum creatinine values converge. The time‐averaged serum water creatinine concentration during the specified urine collection period is used to calculate KrCrW.

For the purpose of this analysis, a new module (KrCrW) of the creatinine kinetic modeling was developed, reversing some of the inputs of the creatinine solver. Instead of the inputs and outputs detailed above, the new model inputs include GCr, VdCr, and predialysis serum creatinine as well as dialysis treatment data. In this new model, KrCrW is not an input but an output. Whereas the original model iterates GCr until the modeled predialysis serum creatinine value matches the measured value, in this new model, an initial “rough guess” value of KrCrW (based on the input predialysis serum creatinine value) is iteratively adjusted until the modeled predialysis serum creatinine value matches the input (measured) value on the day of the modeled dialysis session. For testing purposes, a previously described “what if” creatinine kinetic model [3], which predicts predialysis and postdialysis serum creatinine values based on inputs of GCr, VdCr, KrCrW, and dialysis treatment parameters, was used. The predicted predialysis serum creatinine values from the “what if” model were tested for dialysis schedules ranging from 1 to 7 treatments per week, with test GCr input values of 500, 1200, and 2000 mg/day and test KrCrW inputs of 0, 2.5, 5, 7.5, 10, 15, and 20 mL/min. The predicted predialysis serum creatinine values from the “what if” program were then used as inputs to the new KrCrW module, which, as noted, estimates KrCrW. The KrCrW value from the KrCrW model was compared with the input value for the “what‐if” calculator.

The anthropometric equation used to estimate daily creatinine excretion was published by Ix et al. [7]. This equation is based on a dataset of 2466 subjects. It provides an estimate of the daily creatinine generation rate based on subject age, weight, and gender and also adds a race term of small magnitude, adding 35 mg/day of creatinine generation for subjects of Black race. The equation subtracts 380 mg/day for women. The Ix equation was developed as part of the CKD‐EPI consortium. The mean GFR in the subjects used to develop the equation was 49 mL/min per 1.73 m^2^. Twenty‐five percent of the subjects had a GFR < 30 mL/min. There were no dialysis patients in the dataset used to develop the equation. The equation we used was designated as “Equation D” in their CJASN paper and was as follows, where GCr is the daily creatinine excretion in mg:

GCr = 879.89 + 12.51 × weight(kg) − 6.19 × age +(34.51 if Black) − (379.42 if female)

For the present analysis, we modified the published equation to make it “race neutral” by eliminating the small race term and adding 17 mg/day for all patients, which would result in a 17 mg/day overestimation for Caucasians and a similar underestimate for subjects of the Black race. Given that the average GCr is in the range of 1000 mg/day, elimination of the race term would not be expected to result in a large error.

All patients gave informed consent. The protocol did not change their usual dialysis treatments in any way, except for the collection of urine during the interdialytic interval preceding the modeled dialysis session, and the collection of a predialysis and postdialysis blood sample. All dialysis sessions were given with a blood flow rate of 300 mL/min and a dialysate flow of 500 mL/min with a session length of 240 min. The patients dialyzed once a week were using Fresenius F60S dialyzers, with an estimated in vitro K_0_A urea value of 800 mL/min, and the patients dialyzed twice a week were using a Smartflux L18 dialyzer (Medica Limited Company, Medolla, Italy), for which we estimated the in vitro K_0_A urea value to be 950 mL/min.

## 3 Results

Treatment results are given in Table 1, as calculated by the urea and creatinine versions of Solute Solver. The initial task was to confirm that the KrCrW module was estimating modeled KrCrW values consistent with values obtained from urine collection when the same inputs for GCr, VdCr, and predialysis serum creatinine level were used for both calculations. Using the Creatinine Solver program, the mean value of KrCrW from urine collection was 5.89 ± 3.56 mL/min in the patients dialyzed twice a week, and 8.95 ± 4.04 mL/min in patients being dialyzed once a week.

**TABLE 1:** Dialysis treatment results.

Quantity	Value
UF volume 1/week	0.78 ± 0.79
UF volume 2/week	1.28 ± 0.88
spKt/V urea	1.56 ± 0.13
Kd_urea (mL/min)	196 ± 6.7
KrUrea_w (mL/min)	3.65 ± 1.5
Vd_urea	29.1 ± 2.4
Vd_urea/WatsonV	0.84 ± 0.16
G_urea_nitrogen (mg/min)	4.11 ± 1.0
Predialysis SUN (mg/dL)	67 ± 11
KdCr (mL/min)	140 ± 5.2
KrCrW (meas. from urine collection), mL/min	7.43 ± 4.1
VdCr (mod)	22.7 ± 2.4
VdCr/WatsonV	0.66 ± 0.13
**GCr (mod), mg/day**	**1091 ± 377**
**GCr (ant), mg/day**	**1198 ± 303**
Predialysis serum Cr (mg/dL)	7.39 ± 2.3

*Note:* The table's primary variables of interest are GCr(mod) and GCR(ant), which are highlighted in bold emphases.

Abbreviations: ant = anthropometric; Cr = creatinine; GCr = creatinine generation rate; Kd = dialyzer clearance; Kr = residual kidney clearance; meas. = measured; mod. = modeled; sp = single‐pool; SUN = serum urea nitrogen; UF = ultrafiltration; VdCr = creatinine volume of distribution.

Using the newly developed module, the mean KrCrW values using the modeled inputs for GCr and VdCr were not significantly different: 5.87 ± 3.56 and 8.83 ± 4.00 mL/min, for 2/week and 1/week dialysis patients, respectively.

The remaining modeled values are shown in Table 1.

**FIGURE 1:**
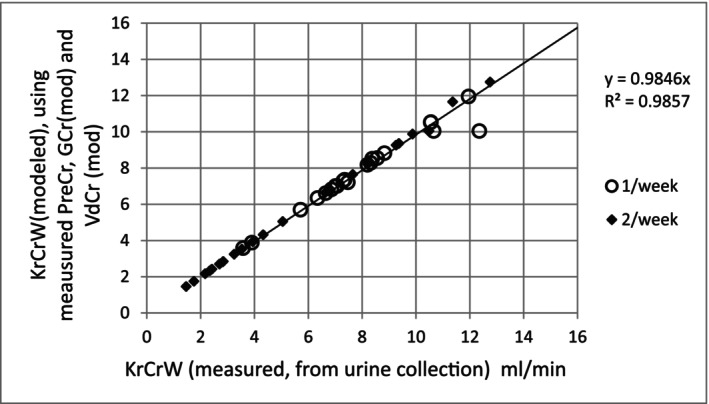
Comparison of predicted residual renal creatinine (water) clearances (KrCrW, vertical axis) with the urine values (horizontal axis) for the same modeling session. PreCr = measured predialysis serum creatinine, GCr (mod) = kinetically modeled value for GCr, VdCr (mod) = kinetically modeled value for 2‐pool creatinine distribution volume.

A scatterplot comparison of the two sets of KrCrW values is illustrated in Figure 1.

Because GCr and VdCr will not be known in clinical use unless they have been determined by a prior modeling session, we explored the utility of substituting the modeled value of GCr with an anthropometric estimate, based on extensive urine creatinine collection by the CKD epidemiology collaboration by Ix et al. [7].

**FIGURE 2:**
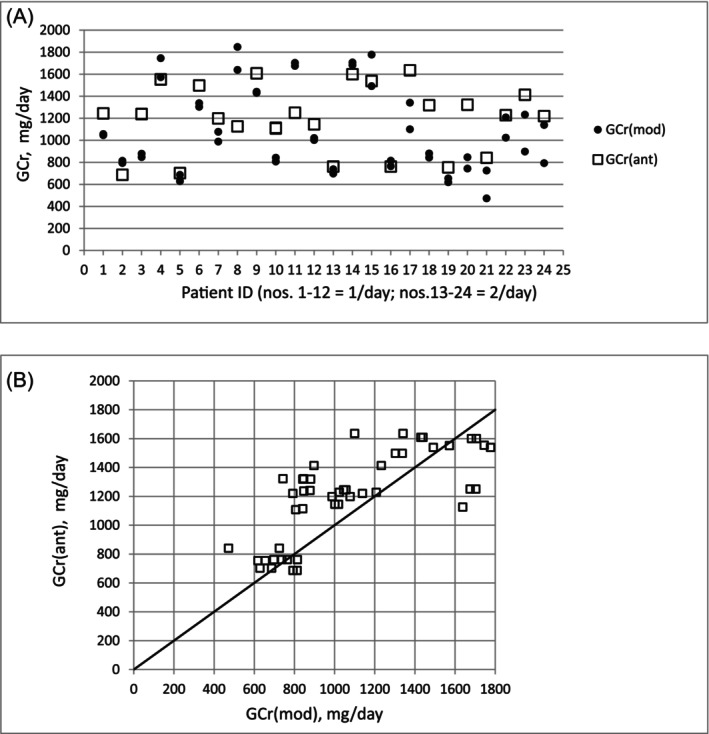
(a) Comparison of an anthropometric estimate of GCr with the modeled value for each of the patients studied. There are two filled circles for each patient, corresponding to calculated values based on long‐duration and 24‐h predialysis urine collections. (b) Scatterplot of an anthropometric estimate of GCr [GCr (ant)] compared with kinetically modeled values [GCr (mod)].

As shown in Table 1, the mean values from the Ix estimate of GCr were similar to the modeled values, although the values predicted by the Ix equation were on average 10% higher (1198 vs. 1091 mg/day). The agreement between the modeled value and the Ix estimate is shown in Figure 2a,b.

**FIGURE 3:**
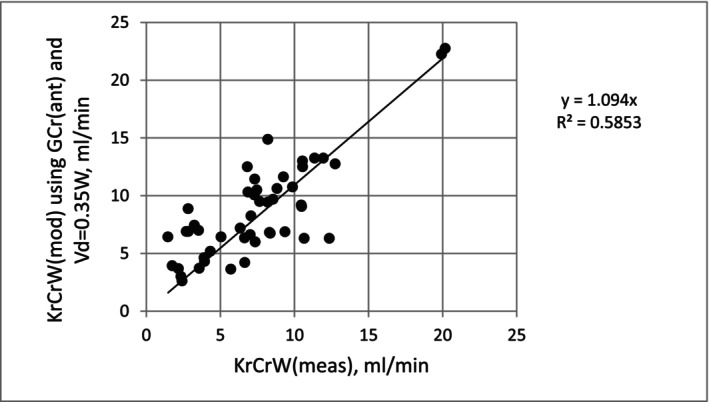
Scatterplot of predicted KrCrW [KrCrW (mod)] on the vertical axis using GCr (ant) and VdCr (ant) as 35% of postdialysis weight vs. the urine‐measured value of KrCrW [KrCrW (meas)] on the horizontal axis.

**FIGURE 4:**
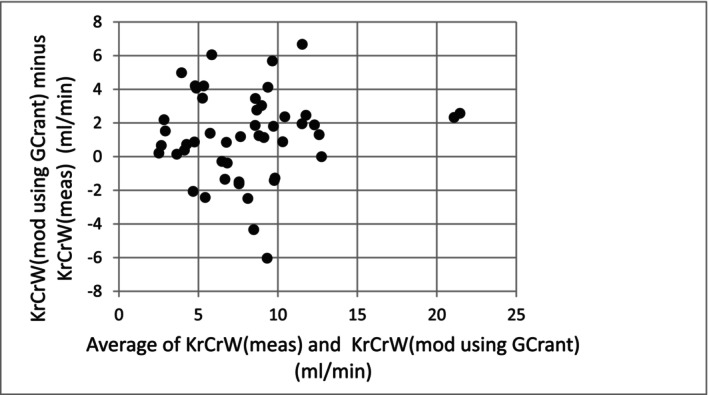
Analysis of error (Bland Altman plot) showing the difference between modeled KrCrW using GCr (ant) and VdCr (ant) minus KrCrW (meas) from collection of urine.

To calculate KrCrW using the new prediction module, one needs to input a patient‐specific value for VdCr in addition to a value for GCr. In the present data set, the mean ratio of VdCr to postdialysis weight was 0.35 ± 0.076. To evaluate the utility of predicting KrCrW from predialysis creatinine and anthropometric values, without knowledge of prior modeled values of VdCr and GCr, we tested the prediction of KrCrW using the Ix estimate of GCr and estimating VdCr as 0.35 × postdialysis weight. The predicted KrCrW was 8.66 ± 4.27 mL/min, somewhat higher than the urine‐based value shown in Table 1 (7.43 ± 4.07). The mean error (Ix minus urine) was 1.23 ± 2.58 mL/min. Agreement between the two methods is illustrated in the scatterplot shown in Figure 3 (*R* ^2^ = 0.58), and the corresponding Bland Altman error plot is shown in Figure 4.

## 4 Discussion

Our findings confirm that using creatinine kinetic modeling, it is possible to come up with an estimate of residual kidney creatinine clearance (KrCrW) that is quite similar to the value obtained using urine collection, provided that correct values for creatinine generation rate (GCr) and creatinine volume of distribution (VdCr) are used along with the measured predialysis serum creatinine value. However, the error in the KrCrW estimate when GCr and VdCr are estimated from anthropometric values is substantial, at least when using only a single session of urine collection and kinetic modeling as the comparator. Ideally, GCr and VdCr and KrCrW should be evaluated across several dialysis sessions, both to check for stability of these values, and also experimental variability. Anthropometric estimates of GCr and VdCr might be more closely associated with averaged values modeled over multiple sessions and instances of urine collection, especially given the problems with urine collection and assay as discussed in the introduction section earlier. One approach to estimating KrCrW serially might be to perform two or three baseline kinetic modeling sessions complete with urine collection and average the resulting values for GCr and VdCr. We have shown that Vd_urea is stable over time in hemodialysis patients in whom the body weight does not change much. The value for Vd_urea also does not change much in patients who gain weight over a 1‐ or 2‐year period, which was interpreted as most of such body weight gain being fat. However, Vd_urea did decrease in patients who had substantial decreases in body weight over time [8].

Creatinine generation is linked to lean body mass, primarily because of the large contribution of muscle mass [9]. In dialysis patients who lose muscle mass over time, lost muscle mass should be reflected by a change in the serum creatinine [10]. In dialysis patients at stable weight in whom the predialysis serum creatinine decreases, another possible explanation might be partial recovery of renal function, which is not uncommon in patients whose renal failure followed an acute kidney injury. Distinguishing a drop in serum creatinine due to loss of lean body mass vs. partial renal recovery should not be difficult clinically.

In those patients at stable weight who begin with substantial residual renal function, an increase in serum creatinine over time most likely indicates loss of residual renal clearance. Identification of loss or renal clearance may be especially important in patients being dialyzed on an incremental (1/week or 2/week) schedule, because it has been shown that when residual renal clearance of urea is below 3 mL/min per m^2^, a variety of adverse outcomes is increased [10], though this is not a universal finding. The current KDOQI adequacy guidelines, although opinion‐based, recommend restricting twice‐a‐week dialysis schedules to patients with residual kidney urea clearances greater than 2.0 mL/min per 1.73 m^2^ [11]. It is recommended to monitor patients using incremental hemodialysis schedules with periodic urine collections, but our data suggest that there may be an alternative. One can perform a pair or three modeling sessions to obtain modeled average GCr and VdCr values, and then use these as inputs with the KrCrW module to track KrCrW over time from routinely measured monthly predialysis serum creatinine values, performing follow‐up urine collections only as needed.

The main limitation of our study is that the potential utility of anthropometric estimates for GCr and VdCr was assessed against a single modeling session as a comparator. The utility of the model in detecting changes in KrCrW over time was not assessed. Finally, dialyzer clearance of both urea and creatinine was estimated from published in vitro K_0_A urea values for the two dialyzers used. One way to test the reliability of a dialyzer clearance estimate is to compare the ratio of modeled Vd_urea or VdCr to an anthropometric estimate of total body water, such as that using the Watson equations. In this analysis, the mean Vd_urea/Watson value (0.84) was as expected, but the mean VdCr/Watson value (0.66) was somewhat lower than previously determined [12] in HEMO study data where cross‐dialyzer clearance was measured rather than estimated. Balanced errors in G and V in kinetic modeling tend to be self‐correcting, so this should not markedly affect the estimation of KrCrW.

One technical point: The models for urea, creatinine, and phosphate at ureakinetics.org calculate residual kidney function as blood or plasma water clearances because the models calculate dialyzer water clearances. To convert to the usually obtained plasma clearances, the KrCrW value needs to be multiplied by 1.075 (assuming 7% plasma protein concentration).

There are other empirically derived equations to predict residual kidney function. One innovative approach to achieve such a goal used values from pre‐ and post‐dialysis bioimpedance measurements (phase angle, resistance, and reactance) as well as predialysis serum creatinine [13]. It would be useful to compare the accuracy of this approach with the present method.

In summary, we present a tool that can be used to estimate KrCrW in patients receiving hemodialysis treatments, as long as baseline values of GCr and VdCr are known. Given that predialysis serum creatinine values are routinely measured monthly in most large dialysis organizations in the United States, application of this tool, in conjunction with analysis of body weight changes, might allow monitoring of residual kidney function over time. This knowledge can impact decisions regarding modification of the dialysis schedule, identify patients who may be experiencing some recovery of renal function, and allow analysis of residual renal function in large dialysis organization datasets to help identify risk factors for renal function decline.

The validation part of the present study is really a comparison of anthropometrically predicted GCr with the modeled value. We are not proposing the model for clinical use without further validation. We believe that averaging and trending predialysis serum creatinine over time would help reduce measurement error.

Our data suggest that the accuracy of an anthropometric estimate of GCr might not be sufficient to reliably estimate KrCrW. If one looks back at the validation of the Ix equation anthropometric estimate (Table 2 on page 187 on the *CJASN* paper by Ix et al.), the P30 value using an external validation data set (P30 is the percent of cases where the anthropometric GCr estimate is within 30% of the measured GCr from urine collection) was 80%. The true agreement might be higher because there is substantial variability in the completeness of urine collection. Such a range might be too wide to be clinically useful in predicting the exact amount of KrCrW, but it may have some utility in trending the amount of residual kidney function.

We believe that the optimal approach might be to collect urine from new patients admitted to a dialysis unit on perhaps two occasions in order to calculate a modeled, patient‐specific GCr value (using our previously published creatinine kinetic model). We believe that, absent a marked decrease in postdialysis body weight over time, the GCr value in dialysis patients will be relatively constant, and when an initially modeled value of GCr is used, then changes in KrCrW can be monitored by simply tracking, averaging, and trending the serum creatinine value. However, the clinical utility of this approach needs to be proven with subsequent studies. The purpose of the present manuscript is to propose a pathway for estimating KrCrW based on the serum creatinine value and the GCr, along with details of the dialysis treatment and the timing of the predialysis serum creatinine sample.

## Ethics Statement

All patients provided informed consent. The protocol did not deviate from the patients' usual treatment, with the exception of urine collection during the interdialytic interval.

## Conflicts of Interest

The authors declare no conflicts of interest.

## Data Availability

Data are available from coauthor Dr. Bolasco upon request.
